# Wave-driven resuspension as the dominant environmental predictor of enterococci at Chicago beaches

**DOI:** 10.1007/s10661-026-15791-3

**Published:** 2026-08-27

**Authors:** Lucy Ferron, Abhilasha Shrestha, Marcia R. Silva

**Affiliations:** 1 Illinois Mathematics and Science Academy (IMSA), Aurora, IL USA; 2 School of Public Health, University of Illinois Chicago (UIC), Chicago, IL USA; 3 AIHealth4All, College of Medicine, University of Illinois Chicago, Chicago, IL USA; 4 Present Address: Office of Research and Innovation, University of Missouri - Kansas City, Kansas City, MO USA

**Keywords:** Beach monitoring, Fecal indicator bacteria, Lake Michigan, SHAP, Random forest, Hydrodynamics

## Abstract

Predicting fecal indicator bacteria (FIB) to protect public health at Chicago beaches remains a significant challenge due to complex, site-specific environmental dynamics. This study presents a machine learning pipeline utilizing site-specific random forest regressor models to predict log-transformed enterococci concentrations. The models integrate meteorological, hydrodynamic, and temporal-lag features. A hybrid evaluation framework is implemented; models are trained for regression but evaluated on their ability to classify bacterial exceedances at a 320 CCE/100 mL threshold, chosen as a conservative value given data sparsity at the Chicago Park District’s Beach Action Value of 1000 CCE/100 mL, which is used to issue swim advisories at these recreational beaches. This is achieved by dynamically identifying an optimal F1 score threshold for each model’s raw count predictions, linking regression performance to classification utility. Model interpretability was a primary focus, employing SHAP (SHapley Additive exPlanations) for each beach and in aggregate. This analysis provided critical insights into the distinct drivers at each location. For the Chicago beaches, the models identified hydrodynamic factors, specifically maximum wave height and maximum dominant wave period, as the most significant hydrometeorological predictors of enterococci concentrations, highlighting the importance of nearshore physical processes in this region. The resulting pipeline provides an interpretable, validated, and site-specific forecasting system capable of identifying high-risk contamination events with moderate accuracy but struggling with sensitivity.

**Supplementary Information:**

The online version contains supplementary material available at https://doi.org/10.1007/s10661-026-15791-3.

## Introduction

Chicago’s beaches are a key recreational resource for tourists and local residents during the summer months. Some of these beaches frequently experience elevated concentrations of fecal indicator bacteria (FIB) in nearshore waters. In the past, the Chicago Park District tested bacterial water quality with *Escherichia coli* (*E. coli*) using culture-based methods for beach monitoring and public notification purposes. Since 2017, the Chicago Park District has transitioned into testing with enterococci using the quantitative polymerase chain reaction (qPCR) method (Shrestha & Dorevitch, 2020). Chicago Park District issues advisories at beaches using the Beach Action Value (BAV) of 1000 CCE which corresponds to estimated illness rate (NGI) of 36 per 1000 primary contact recreators (Chicago Park District, n.d.). Elevated levels of enterococci suggest that the water may be contaminated with fecal matter, which increases the likelihood that harmful pathogens such as bacteria, viruses, and protozoa could also be present. Exposure to water contaminated with these harmful pathogens can lead to health issues such as gastroenteritis and infections, and contact with the beach sand itself has been shown to increase the risk of enteric illness among beachgoers (Fleisher et al., 2010; Heaney et al., 2012). Previous studies have identified environmental drivers of elevated bacterial concentrations. Some key predictors of bacterial concentrations are precipitation (Ackerman & Weisberg, 2003), temperature (Samspon et al., 206), wind speed and direction (Smith et al., 1999), and wave height (Le Fevre & Lewis, 2003). Close proximity to combined sewer overflow (CSO) events has also been shown to increase bacterial concentrations at beaches (McLellan et al., 2007). However, since the Chicago River flows away from Lake Michigan, CSOs do not typically influence microbial water quality at Chicago beaches (Steele, 2025). Key nonpoint sources of contamination at Chicago beaches include pollution linked to bird and dog activity (Shrestha et al., 2020) and bacteria from foreshore sand (Whitman & Nevers, 2003).

Research on bacterial trends at Chicago beaches in recent years has been limited, but older studies found that Julian day, wave height, and barometric pressure explained up to 40% of the variation at 23 Chicago beaches and that beaches within a closer spatial proximity had higher correlations in bacterial concentrations (Whitman & Nevers, 2008). Diurnal variation of *E. coli* concentrations has also recently been observed at 63rd Street Beach in Chicago (Boehm, 2007). Some recent work has focused on building predictive models using inter-beach qPCR data correlations (Lucius et al., 2018). Another Chicago study analyzing nine Chicago beaches found that the adjusted R^2^ relating *E. coli* densities and hydrometeorological variables were greater for empirical models than for persistence models (Shively et al., 2016). Jones et al. (2012) used tree regression and random forests to select the most important hydrometeorological variables at Chicago beaches, demonstrating that simpler linear models built with these few selected features could predict bacterial densities with reasonable accuracy, thereby offering a cost-effective alternative to resource-intensive monitoring but suffered from limited sensitivity in predicting high-risk events. More recently, Elahi et al. (2025) found that transfer learning (TL) improved machine learning models developed at data-rich San Diego beaches when applied to Chicago beaches.

Predictive models in Chicago can support beach management due to their ability to anticipate bacterial spikes based on incoming hydrometeorological data. A combination of different monitoring methods maximizes beach access without causing public health risks (Nevers & Whitman, 2011).

Despite decades of monitoring bacteria levels at Chicago beaches, there has been relatively little research in the past decade that examines recent trends in bacterial concentrations specifically at Chicago beaches. The scarcity of recent Chicago-focused studies hinders the ability of beach management to understand trends, as most of the available research focused on Chicago predates significant changes in monitoring technology and does not make use of bacterial data from the past decade. The goal of this manuscript is to help academic researchers understand recent bacterial patterns and to develop predictive models for bacterial levels at each beach. The methodologies and interpretability frameworks developed here are intended to be transferable to other Great Lakes or coastal environments where wave-driven resuspension is suspected to be a primary driver of increased bacterial concentration.

## Materials and methods

### Study area

**Fig. 1:**
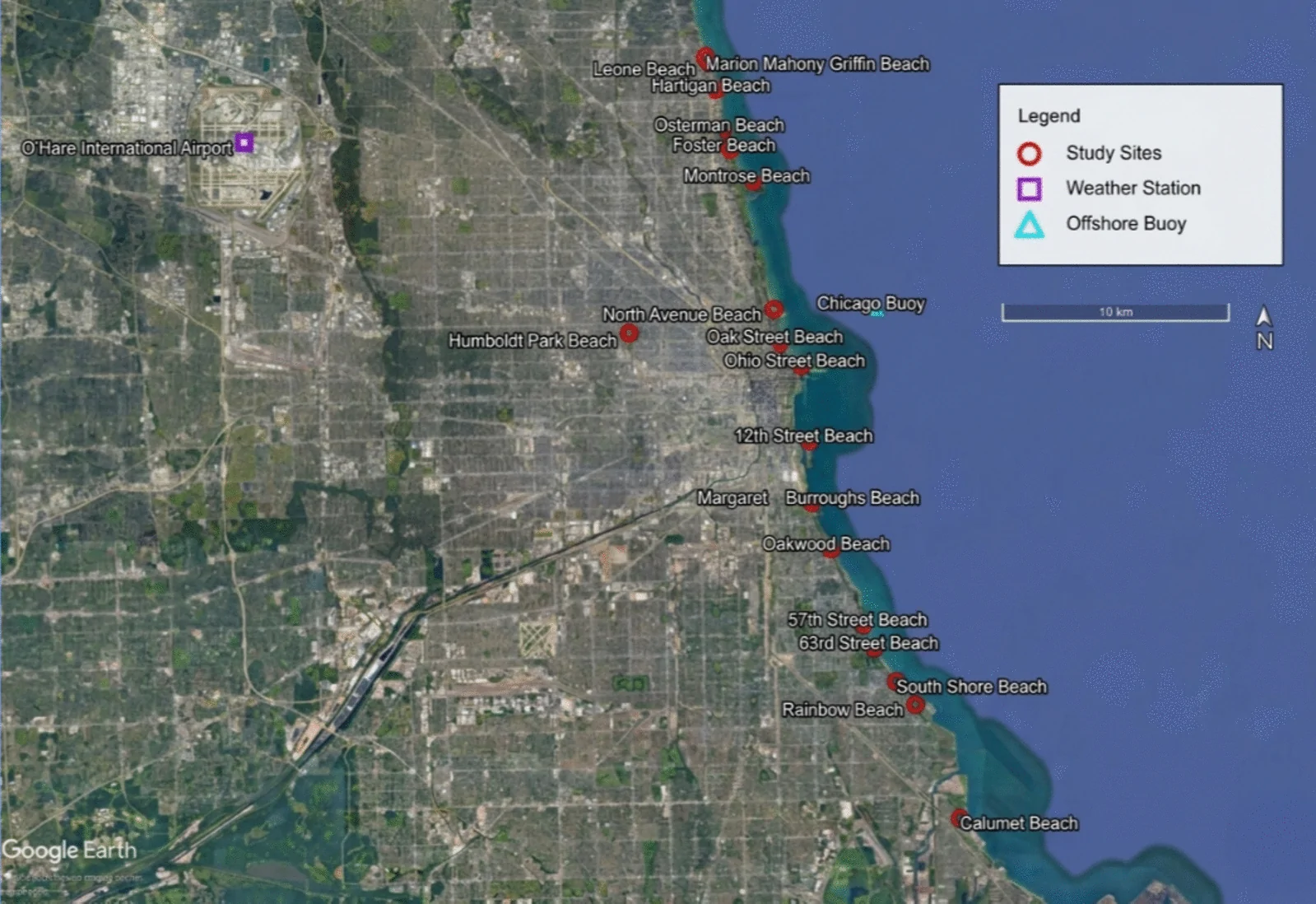
Study area map of the 18 enterococci monitoring beaches along the Chicago Lake Michigan shoreline. Created using Google (n.d.) Map created using Google Earth Pro (Google LLC); imagery accessed 7 Jul 2026

The study area, illustrated in Fig. 1, comprised 17 Lake Michigan beaches and one inland beach (Humbold Park Beach) in Chicago. These locations were selected based on the availability of enterococci concentration data, which were routinely collected as a part of a local water quality monitoring program conducted by the Chicago Park District in partnership with the University of Illinois at Chicago Department of Public Laboratory (City of Chicago, n.d.). The locations of Chicago O’Hare International Airport and the Chicago Buoy are also shown in Fig. 1.

### Bacterial data collection

Enterococci concentrations were obtained from the City of Chicago Beach Lab Data with measurements reported as calibrator cell equivalents (CCE) per 100 mL based on qPCR analysis conducted by the University of Illinois Chicago School of Public Health (City of Chicago, n.d.). Chicago beaches are seasonally open from late May through early September, and daily bacterial water quality monitoring is conducted during this period (University of Illinois Chicago, School of Public Health, 2026). For this study, only data from 2021 onward were included, as 2021 was the first year with concurrent wave data available from the NOAA National Buoy Center (Station 45198, Chicago Buoy), which were used as environmental predictors in the model. The distance between the buoy and the beaches varied from approximately 4.5 miles to over 12 miles for the southernmost beaches.

### Hydrometeorological data collection

Wave height, wave period, air temperature, and sea surface temperature were obtained from the National Oceanic and Atmospheric Administration (NOAA) National Data Buoy Center (NDBC) (Station 45198, Chicago Buoy) (NDBC, n.d.). As these variables were reported every 10 min, maximum values for each 24-h period were selected. Additional meteorological variables of precipitation, average wind speed, direction of fastest 5-s wind, and direction of fastest 2-min wind were obtained from the National Center for Environmental Information (NCEI) weather station at Chicago O’Hare International Airport (NCEI, n.d.).

### Software

All analyses were conducted in Python (v3.12.12) using pandas (v2.3.3) for data handling, NumPy (v2.0.2) for numerical computations, scikit-learn (v1.6.0) for random forest regression modeling and evaluation, and SHAP (v0.48.0) and Matplotlib (v3.10.0) for interpretability and visualization.

### Model

The Claude 4.5 Sonnet large language model (Anthropic) was utilized to assist in the development, optimization, and debugging of the Python-based data processing scripts and the random forest modeling pipeline. The final code and resulting outputs were manually reviewed and verified by the authors to ensure accuracy.

Enterococci concentrations were modeled separately for each beach using a random forest regression approach. Predictor variables were derived from hydrometeorological, temporal, and lagged bacterial measurements. The full list of predictors included the following:

- Maximum wave height (ft)
- Maximum dominant wave period (s)
- Maximum air temperature (°C)
- Maximum lake surface temperature (°C)
- Precipitation (in)
- Average wind speed (mph)
- Direction of fastest 5-s wind (°) (decomposed into sine and cosine components)
- Direction of fastest 2-min wind (°) (decomposed into sine and cosine components)
- Day of the year (Julian day)
- Month
- Precipitation 24-h lag
- Precipitation 48-h lag
- 72-h precipitation total
- Any rain in previous 24 h (binary)
- Interaction between sea surface temperature and 24-h lagged precipitation
- Log-transformed 1-day lag of enterococci concentration at the same beach

The inclusion of a 1-day lag of enterococci concentration as a predictor is made operationally feasible by the specific monitoring infrastructure in Chicago. The qPCR-based testing provided a median turn-around time of 3 h and 59 min in 2019, with results typically reported by midday on the day of collection (Shrestha & Dorevitch, 2020). Because Chicago’s qPCR results are ready by midday, the previous day’s data is available to use in the model the following morning.

The model incorporates an interaction term between lake surface temperature (LST) and 24-h lagged precipitation. This feature was included to account for the relationship between storm-driven runoff and thermal conditions. While precipitation events transport bacteria into the nearshore zone, warmer water temperatures facilitate the persistence of these bacteria. This specific interaction is a documented driver of bacterial spikes in temperate coastal environments (Sampson et al., 2006; Whitman & Nevers, 2003).

For Humboldt Park Beach, an inland location, wave-related predictors were excluded from the model feature set as these parameters are not applicable to noncoastal environments. To address the right skewness of the bacterial count data and stabilize variance, enterococci concentrations were log transformed using the log1p function defined as ${y}{\prime}=loglog \left(x+1\right)$. Units were left unchanged during data treatment and no data points were removed.

To prevent data leakage, a chronological data splitting strategy was implemented. For each beach, the first 80% of the temporal record was designated as the training set, while the final 20% was reserved as an independent test set (shuffle = False). This process ensured that the model demonstrated true predictive capability rather than memorization of temporal patterns (Zhu et al., 2023). Within the training phase, a nested 80/20 split was further applied to create a hold-out validation set, which was used to dynamically identify the optimal site-specific F1 score threshold.

The random forest regressor was tuned using a threefold TimeSeriesSplit cross-validation, which uses an expanding window approach to maintain the temporal order of observations. The optimization objective was negative root mean squared error (RMSE). The hyperparameter grid included the number of trees (100, 250, or 400), maximum tree depth (10, 20, or unrestricted), and the minimum number of samples required at a leaf node (1 or 5).

Random forest hyperparameters were tuned using a constrained grid search with time series cross-validation, optimizing negative root mean squared error to balance predictive performance. The number of trees, maximum depth, and minimum leaf size were varied to control model complexity and reduce overfitting. The hyperparameter space was intentionally limited to preserve model stability.

### Model evaluation criteria

In Chicago, the Park District issues swim advisories at a Beach Action Value (BAV) of 1000 CCE/100 mL, which corresponds to an estimated illness rate (NGI) of 36 per 1000 primary contact recreators (CPD, n.d.). While the U.S. Environmental Protection Agency (EPA) also defines a conservative BAV of 640 CCE/100 mL (NGI of 32 per 1000 primary contact recreators), the current study used a lower research threshold of 320 CCE/100 mL for model evaluation (U.S. EPA, 2012). This decision was based on the methodology aligned by Elahi et al. (2025). Predicted concentrations were exponentiated back to original scale, and dynamic optimal thresholds were determined for each beach to maximize F1 score in classifying predicted exceedances relative to observed exceedances. Model performance metrics included the following:

- F1 score, precision, sensitivity, and balanced accuracy
- Receiver operating characteristic–area under the curve (ROC AUC)

The F1 score is defined as the harmonic mean of precision and sensitivity, providing a balanced measure of a model’s accuracy by accounting for both false positives and false negatives. The performance of the model was evaluated using a binary classification framework based on the 320 CCE/100 mL threshold. The following outcomes were defined:

- True positives (TP): Instances where both the model and the observed water samples identified an exceedance
- True negatives (TN): Instances where both the model and the observations identified safe water quality
- False positives (FP): Predictions of an exceedance that were not observed in the water samples
- False negatives (FN): Predictions of safe water quality samples indicated an when water exceedance

To quantify predictive utility, the following performance metrics were calculated:

- Accuracy = $$\frac{TP+TN}{TP+TN+FP+FN}$$
- Sensitivity = $$\frac{TP}{TP+FN}$$
- Specificity = $$\frac{TN}{TN+FP}$$ 
- F1 Score = $$2\bullet \frac{Precision \bullet Recall}{Precision+Recall}$$

Area under the receiver operating characteristic curve (AUC) was also calculated. AUC quantifies the ability of a binary classifier to distinguish between positive and negative cases across all possible thresholds. It is formally defined as the area under the receiver operating characteristic (ROC) curve which plots the sensitivity against the false positive rate (1, specificity) for all thresholds:

$$AUC= {\int}_{0}^{1}TPR\left(FPR\right)d(FPR)$$

where $$TPR= \frac{TP}{TP+FN}$$ and $$FPR= \frac{FP}{FP+TN}$$.

Interpretability analyses were performed using SHAP values to quantify the relative importance and effect of individual predictors on predicted enterococci concentrations. SHAP values quantify the magnitude and direction of each predictor’s influence on the log-transformed enterococci concentration for every individual observation. In this study, SHAP summary plots were used to rank the global importance of predictors.

## Results

### Individual model performance

The predictive performance of the random forest model varied across the individual beach sites included. Table 1 lists the classification statistics for each beach model.

**Table 1:** Per beach model performance for random forest model

Beach name	Accuracy	Sensitivity	Specificity	AUC	Total exceedances
Marion Mahony Griffin Beach (*n* = 437)	0.70	0.57	0.74	0.75	84
Leone Beach (*n* = 439)	0.79	0.58	0.85	0.80	82
Hartigan Beach (*n* = 438)	0.86	0.11	1.00	0.69	50
Osterman Beach (*n* = 438)	0.81	0.40	0.85	0.78	49
Foster Beach (*n* = 434)	0.88	0.00	1.00	0.80	38
Montrose Beach (*n* = 434)	0.46	0.86	0.33	0.67	101
North Avenue Beach (*n* = 432)	0.96	0.50	1.00	0.72	30
Oak Street Beach (*n* = 431)	0.48	1.00	0.44	0.87	36
Ohio Street Beach (*n* = 437)	0.63	0.38	0.84	0.68	85
12th Street Beach (*n* = 435)	0.65	0.76	0.60	0.72	55
Humboldt Park Beach (*n* = 171)	0.96	0.00	1.00	0.63	56
Margaret T. Burroughs Beach (*n* = 427)	0.71	0.06	0.97	0.56	107
Oakwood Beach (*n* = 428)	0.85	0.33	0.88	0.54	42
57th Street Beach (*n* = 432)	0.68	0.00	0.73	0.22	36
63rd Street Beach (*n* = 426)	0.31	0.93	0.08	0.58	112
South Shore Beach (*n* = 437)	0.52	0.67	0.49	0.61	76
Rainbow Beach (*n* = 438)	0.90	0.43	0.96	0.76	61
Calumet Beach (*n* = 434)	0.65	0.57	0.69	0.60	110

### Aggregate model performance

To summarize model performance across all 18 beaches, both macro-averaged and micro-averaged metrics were calculated.

Micro-averages, calculated from the total counts across all beaches, provided an overall performance measure. From these counts, overall micro-averaged metrics for the model with hydrodynamic features were as follows:

- Accuracy 0.70
- F1 score 0.40
- Sensitivity 0.51
- Specificity 0.75

The overall micro-averaged metrics without hydrodynamic features were as follows:

- Accuracy 0.68
- F1 score 0.27
- Sensitivity 0.43
- Specificity 0.70

**Fig. 2:**
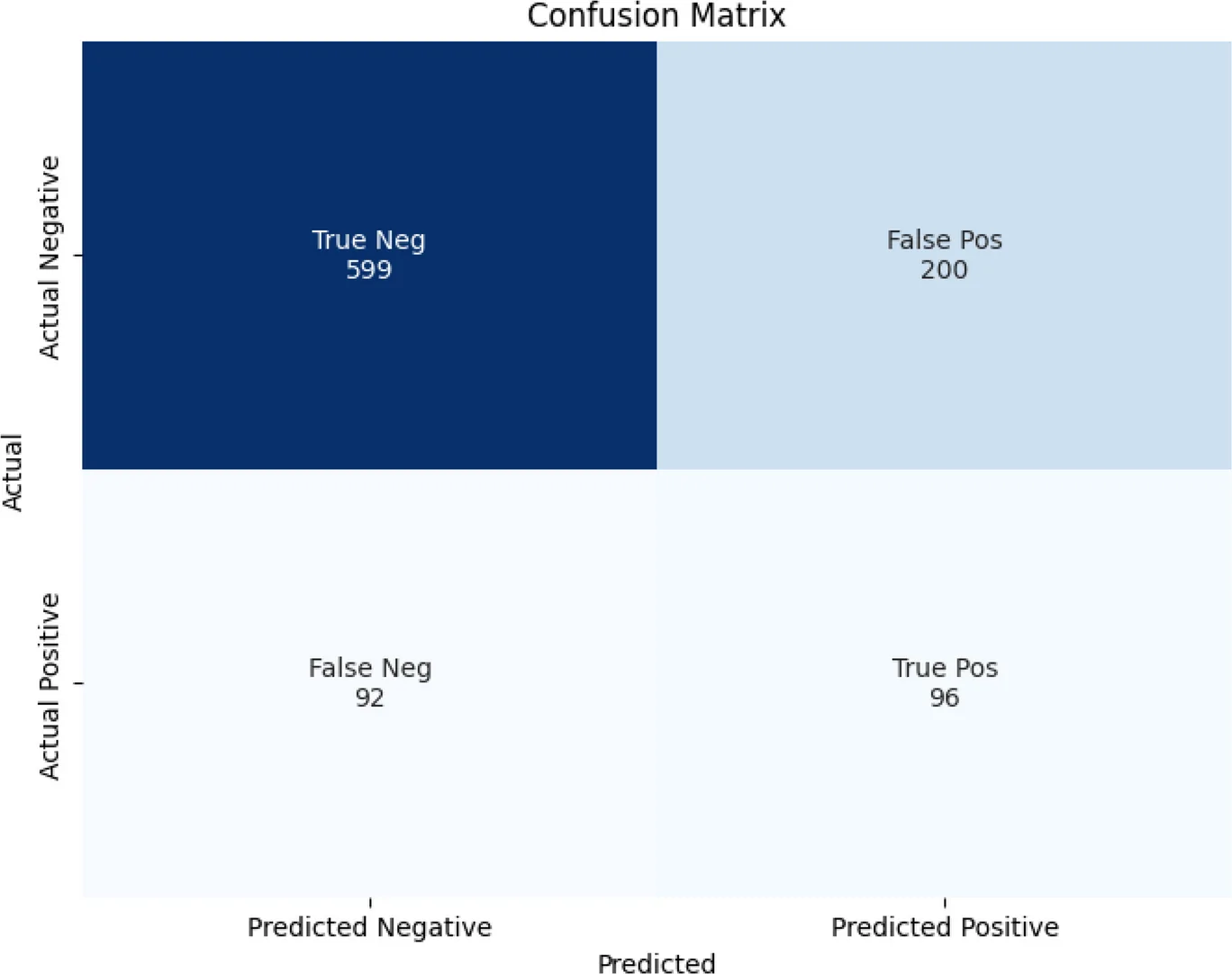
Confusion matrix for the calculation results (N=987)

The performance of the model is summarized in the confusion matrix shown in Fig. 2.

**Fig. 3:**
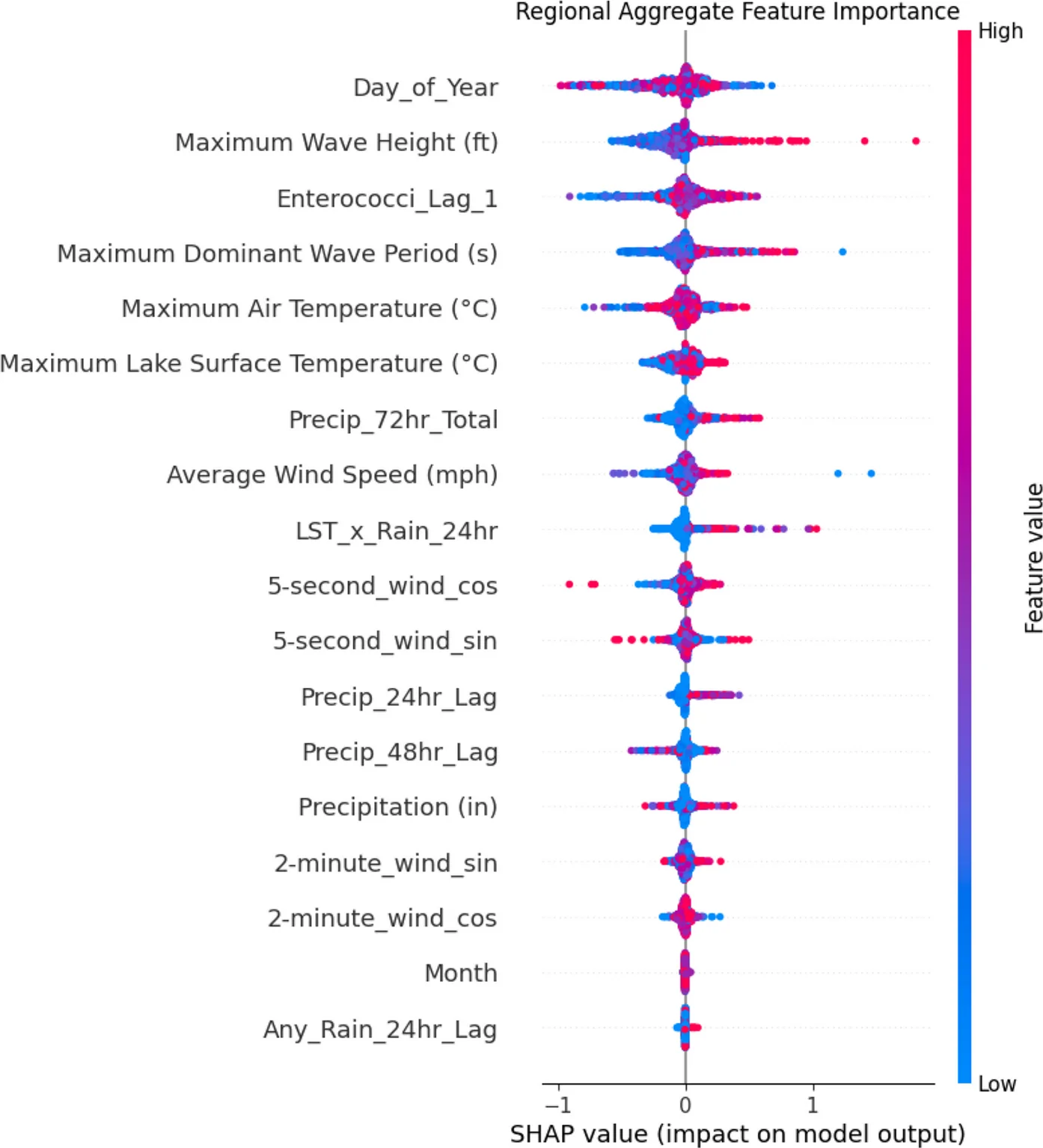
SHAP summary plot showing the relative importance of top model predictors

Figure 3 shows the SHAP summary of the strongest model predictors.

The SHAP analysis that in aggregate, the features with the highest impact were day of the year, maximum wave height (ft), 24-h lagged enterococci concentrations, maximum dominant wave period (s), and maximum air temperature (°C) Per beach SHAP plots are available in the supplementary materials (Figures S1 – S18).

**Fig. 4:**
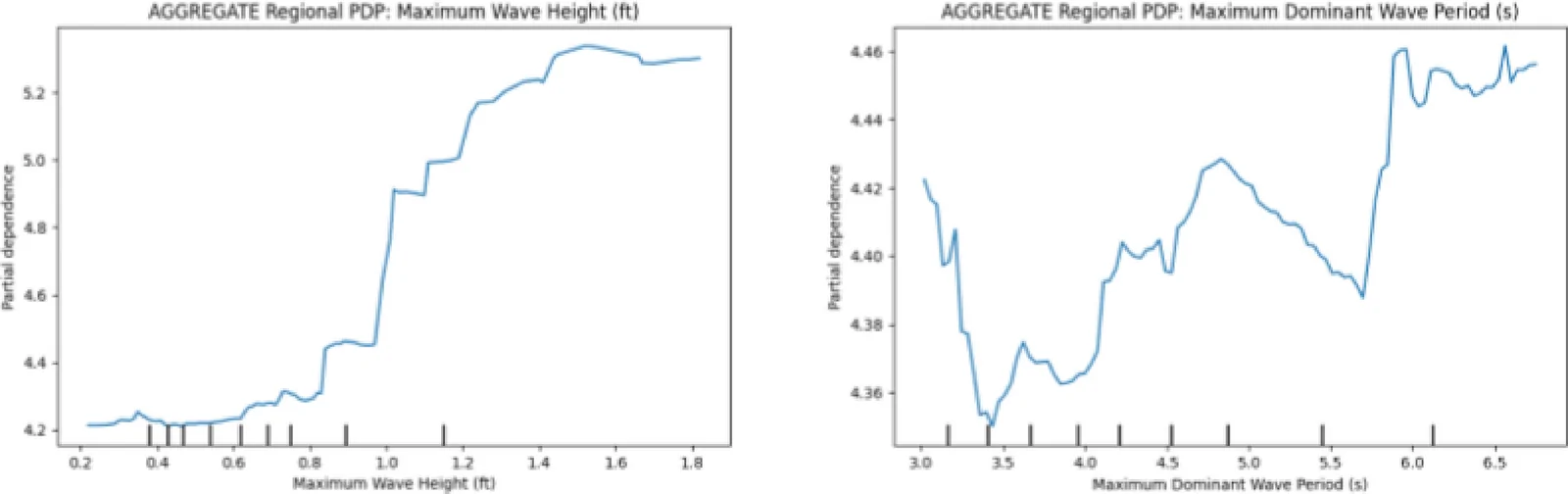
Aggregate partial dependence of enterococci levels on wave dynamics

Figure 4 illustrates the partial dependence of enterococci concentrations on wave dynamics.

The PDP for maximum wave height revealed a strong positive correlation. Conversely, the maximum dominant wave period exhibited a nearly horizontal profile.

### Continuous model dynamics: north avenue beach case study

**Fig. 5:**
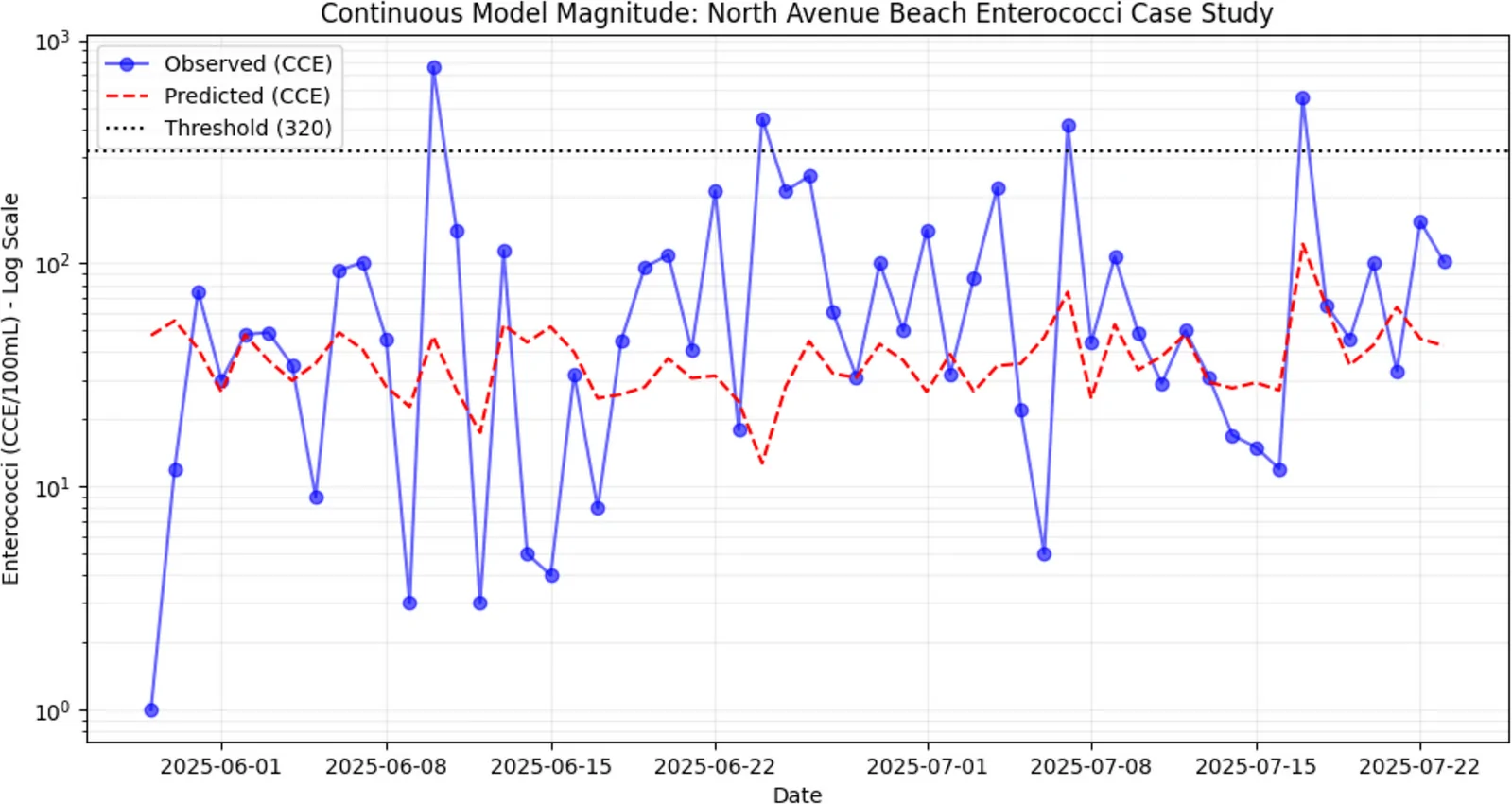
Time series comparison of observed vs. predicted enterococci concentrations at North Avenue Beach

To better evaluate the utility of the continuous predictive output, a case study was conducted at North Avenue Beach (Fig. 5). This site-specific analysis illustrates how the model tracks the magnitude of enterococci concentrations over time.

## Discussion

These results indicate that the model is moderately capable of identifying high-bacteria days. Overall, the random forest regression hybrid approach demonstrates moderate predictive capability for classifying exceedances.

This study addresses a noted gap in recent Chicago-focused beach research, much of which predates the widespread adoption of qPCR monitoring methods (Shrestha & Dorevitch, 2020). By developing a modern, per-beach machine learning pipeline, our work builds upon recent efforts (Lucius et al., 2018) to provide managers with a functional predictive tool. The model demonstrated moderate predictive performance.

The most critical finding from the interpretability analysis (Fig. 2) was the overwhelming and consistent importance of hydrodynamic factors, specifically maximum wave height and maximum dominant wave period. The SHAP analysis revealed heterogeneity in predictor importance across the 18 beaches. Despite this site-specific variability, maximum wave period, maximum wave height, and 24-h lagged enterococci concentration consistently ranked among the most dominant predictors. This finding provides strong, quantitative validation for a key physical mechanism driving contamination at these beaches. Earlier research identified foreshore sand as a significant reservoir for bacteria that poses a risk to public health through sand contact (Heaney et al., 2012; Whitman & Nevers, 2003). Our model’s strong reliance on wave height suggests that wave-induced turbulence is the primary engine for resuspending these sequestered bacteria from the sand into the water column, leading to the observed elevated levels of enterococci concentrations. This result empirically confirms the link between wave action, identified in older Chicago studies (Whitman & Nevers, 2008; Le Fevre & Lewis, 2003), and the specific nonpoint pollution source of contaminated sand. While other predictors like precipitation (Ackerman & Weisberg, 2003) were locally important, the dominance of wave height pinpoints nearshore physical processes as the central driver for contamination in this system.

Wave action is a critical factor in this model because it controls the motion at the shoreline. This relationship is supported by Phillips et al. (2014), who used a wave flume to show that waves can physically pull about 60% of bacteria out from sand and into the surrounding. This study found that this process occurs with a steady release of bacteria occurring in the first 6 min of wave activity. Specifically, waves were more effective at clearing the top layer of sand (78% removal) than the deeper layers (58%). These findings explain why wave height is a strong predictor in the model; it measures the force available to wash enterococci out of the beach sand. The model’s reliance on these hydrodynamic features reflects the predictive signature of the mass transport dynamics explored by Ge et al. (2012) and Silva (2013). Specifically, Ge et al. (2012) established that wave-induced mass transport is the fundamental driver of daily indicator bacteria fluctuations in nearshore waters.

While the 24-h maximum wave height was a dominant predictor, the specific timing of wave peaks relative to the sampling window may introduce some unexplained variance. Chicago’s nearshore environment exhibits an environmental “memory” effect, where elevated bacterial concentrations persist following high-energy events (Whitman & Nevers, 2008); however, the precise rate of this dissipation is not fully captured by a static daily maximum. Future modeling efforts could refine this relationship by incorporating short-term antecedent wave activity (wave activity in the 3–6 h immediately preceding sample collection) to more accurately weigh wave energy based on its proximity to the sampling event.

The four false negatives identified in the North Avenue Beach case study (Fig. 5) show a nuance in the hybrid model’s dynamics. The continuous plot demonstrates that the model can match temporal trends but frequently misses the peak magnitude of extreme events. These missed magnitudes suggest that the pipeline may be missing highly localized factors. While the model relies on regional, broad environmental predictors like offshore buoy hydrodynamics and airport weather data, extreme localized spikes can be driven by transient, micro-scale features. For example, a sudden influx of birds or heavy dog activity directly on the shoreline can cause immediate, massive spikes in enterococci that do not correlate with wave energy or precipitation.

Furthermore, the SHAP analysis revealed substantial heterogeneity in predictor importance across the 18 beaches. This finding underscores the necessity of the per-beach modeling approach. It also provides a modern context for older findings, such as the observation that empirical models could outperform predictive ones when models were not sufficiently localized (Shively et al., 2016).

At Northern sites such as Leone Beach and Osterman Beach, the SHAP analysis revealed that maximum wave period and wave height were the primary predictors. The high importance of these variables suggests that these beaches are highly susceptible to wave-driven resuspension, possibly due to the northern shoreline’s direct exposure to the N/NE fetch of Lake Michigan where wind energy can generate high-energy and long-period waves.

A significant shift in the model behavior occurs at central beaches near downtown Chicago like North Avenue Beach. In these locations, the day of the year and the lake surface temperature emerge as strong predictors, suggesting that at these high-attendance beaches, bacterial concentrations are more closely linked to seasonal cycles than hydrometeorological events.

In the southern reaches of the study area, the influence of wave action diminishes. At Rainbow Beach and 63rd Street Beach, 24-h lagged enterococci concentration and precipitation variables move higher in the SHAP rankings. This geographic variation is likely due to the specific coastal geometry and artificial breakwaters present at these sites. Furthermore, these southern beaches are the furthest from the Chicago Buoy (Station 45198). It is possible that the offshore wave height and period measurements do not accurately represent the nearshore hydrodynamics at these distant locations, potentially contributing to the lower observed importance of wave-driven resuspension in the southern models.

The divergent results between the SHAP importance and the PDP for wave period suggest a nonlinear relationship. While the PDP indicates a flat aggregate marginal effect, the high SHAP values imply that wave period provides significant local predictive power, potentially through interactions with wave height. This suggests a complex, nonlinear relationship. The global PDP indicates a flat aggregate marginal effect across the entire dataset, but the high SHAP values imply that wave period provides significant local predictive power under specific environmental conditions. Global averages obscure these relationships because nearshore sediment resuspension is a threshold-dependent physical process; consequently, aggregate metrics smooth over the brief, high-energy wave events that actually drive episodic bacterial spikes (Silva, 2013). This structural limitation, where global averages mask conditional dynamics in complex environmental datasets, highlights an interpretability pitfall when analyzing feature interactions with partial dependence curves (Zhu et al., 2023).

This behavior points to a hydrodynamic interaction effect with wave height. In nearshore physical processes, a long dominant wave period combined with high wave height fuels widespread sediment resuspension. Conversely, a long wave period during low wave height conditions results in negligible physical disturbance of sediments. This threshold-dependent process relies heavily on bed shear stress. Coastal dynamics demonstrate that sediment resuspension is a binary event, remaining dormant until the combined energy of the wave field breaches a critical shear stress threshold, which is typically found near 0.06 Pa in these nearshore environments (Ge et al., 2012). Because the PDP averages the marginal effect across all samples, the powerful impact of wave period during high-energy events is mathematically washed out by the abundance of low-energy days.

Physically, this indicates that maximum dominant wave period acts as an environmental accelerator rather than a standalone linear driver. A long maximum dominant wave period maximizes the resuspension capabilities of waves only after a critical wave height threshold has cleared the initial sand layers, as supported by the flume dynamics described by Phillips et al. (2014).

By building site-specific models, we can account for this local variability, which would be obscured by a single, generalized model. Table 2 shows a comparison of different predictive beach models, predictors used, and associated metrics.

**Table 2:** Comparative performance and methodology of predictive models for fecal indicator bacteria (FIB) exceedance at Chicago beaches

Study citation	Model type	Predictor variables	Sensitivity	Specificity	Prediction accuracy	AUC	Included wave height
Jones et al. (2012)	Tree regression and random forests to select variables and linear mixed effects (LME) models for prediction	Rainfall, lagged FIB, turbidity	23–62%	Not explicitly reported	72–77%	Not explicitly reported	No
Lucius et al. (2018)	Hybrid nowcast (random forest with K-means clustering)	Same-day qPCR at 5 feature beaches	11.2%	98.0%	92.6%	0.753	No
Elahi et al. (2025)	Random forest with transfer learning (TL)	Wave height, water temperature, air temperature, wind speed, wind direction, precipitation, total prior-day solar direct normal irradiance, lagged FIB, tide data	76.0%	68.3%	Not explicitly reported	0.693	Yes
This study	Random Forest	Wave height, wave period, air temperature, lake surface temperature, precipitation, wind speed, wind direction, Julian date, interaction between sea surface temperature and 24-h lagged precipitation, lagged FIB	51%	75%	70%	0.67	Yes

Several limitations warrant consideration. The model relies on environmental data from a single offshore buoy and a single airport weather station, which may not fully capture the micro-scale hydrometeorological conditions at each site-specific beach. A limitation of this study is the lack of direct sunlight (UV) data, which is a major cause of bacterial death (Whitman & Nevers, 2003). In the future, including a consistent source of UV data would potentially improve model performance. Additionally, the model’s accuracy remains moderate (70%), reflecting the inherent, complex variability in microbial water quality.

Future work could enhance model performance by integrating additional site-specific predictors to better quantify the nonpoint sources mentioned in the literature, such as bird activity (Kleinheinz et al., 2006) or direct measurements of bacteria in the foreshore sand itself (Whitman & Nevers, 2003). Despite these limitations, this pipeline provides an interpretable, validated, and site-specific forecasting system that not only identifies high-risk events but also provides strong evidence that wave-driven resuspension is a key contamination pathway at Chicago beaches.

## Conclusion

This study developed a site-specific machine learning pipeline to analyze enterococci concentrations across 18 Chicago beaches, utilizing a random forest architecture and SHAP interpretability analysis. The model’s moderate accuracy and sensitivity suggest it is best suited as an early warning system. The model can provide immediate warnings to beach managers while remaining within a broader framework of traditional laboratory-based monitoring. However, the primary value of this research lies in its interpretability framework, which successfully moves beyond “black-box” predictions to identify the physical mechanisms driving local water quality.

The SHAP analysis provided consistent, quantitative evidence that hydrodynamic factors—specifically maximum wave height and dominant wave period—are the most dominant predictors of bacterial levels in this system. This confirms that wave-driven resuspension of bacteria from foreshore sand serves as the central contamination pathway for the Chicago shoreline. Furthermore, the high degree of heterogeneity observed in predictor importance across different sites underscores the necessity of site-specific modeling over generalized regional approaches.

By linking machine learning outputs to physical nearshore processes, this study provides a validated framework for understanding contamination drivers. Future work should focus on improving model sensitivity by integrating high-resolution, site-specific biological data, such as avian counts or direct measurements of bacteria within the sand reservoir. As an analytical tool, this pipeline offers a modern, transparent method for researchers and stakeholders to diagnose the environmental conditions that lead to poor water quality at urban beaches.

## Supplementary Information

Below is the link to the electronic supplementary material.

ESM 1 ZIP (1.73 MB)

## Data Availability

The bacterial and hydrometeorological datasets analyzed during the current study are available in the City of Chicago Data Portal (Beach Lab Data) at https://data.cityofchicago.org/ and the NOAA National Data Buoy Center (Station 45,198) at https://www.ndbc.noaa.gov/.
